# Clinical and Somatosensory Correlates of Persistent Zoster-Associated Pain at One Month: A Prospective Multicenter Cohort Study

**DOI:** 10.3390/jcm15197494

**Published:** 2026-09-26

**Authors:** Eda Özsezen, Bahadır Aslancan, Ömer Karakoyun, Nur Ecer, Özlem Devran Gevher, Kıvılcım Çınkır Özsaraç

**Affiliations:** 1 Department of Neurology, Şanlıurfa Training and Research Hospital, 63250 Şanlıurfa, Türkiye; edaozsezen@gmail.com; 2 Department of Dermatology and Venereology, Şanlıurfa Training and Research Hospital, 63250 Şanlıurfa, Türkiye; 3 Department of Dermatology and Venereology, Gazi Yaşargil Training and Research Hospital, 21070 Diyarbakır, Türkiye; 4 Department of Dermatology and Venereology, Şanlıurfa Mehmet Akif İnan Training and Research Hospital, 63040 Şanlıurfa, Türkiye; 5 Department of Dermatology, Medical Point Gaziantep Hospital, 27584 Gaziantep, Türkiye

**Keywords:** herpes zoster, zoster-associated pain, neuropathic pain, pressure pain threshold, quantitative sensory testing, postherpetic neuralgia, prognosis, pressure algometry

## Abstract

**Background/Objectives**: Persistent pain after acute herpes zoster (HZ) carries substantial functional burden, but early markers of pain persistence remain uncertain. In an exploratory analysis, we examined whether baseline clinical features and a simple affected-to-contralateral pressure-pain threshold (PPT) ratio were associated with persistent zoster-associated pain approximately one month after rash onset. **Methods**: In this prospective multicenter observational cohort, 117 adults with acute HZ (mean age 58.7 ± 14.7 years, 60.7% women, 18.8% with diabetes mellitus, 16.2% immunosuppressed, 58.1% thoracic dermatome) were assessed 1–7 days after rash onset with clinical variables, the Douleur Neuropathique 4 (DN4) questionnaire, bedside sensory examination and standardized pressure algometry. Two equally reported outcome definitions were applied at follow-up: average and worst 24 h numerical rating scale (NRS) pain ≥ 3. Firth penalized logistic regression was used, with bootstrap internal validation that repeated the predictor-selection step within each resample. **Results**: Of 117 participants, 109 (93.2%) completed follow-up at a mean of 29.8 ± 3.3 days. Average 24 h NRS ≥ 3 occurred in 25/109 (22.9%) and worst 24 h NRS ≥ 3 in 42/109 (38.5%). Under the average-pain definition, age per 10 years (odds ratio [OR] 1.60, 95% confidence interval [CI] 1.03–2.59), baseline NRS per point (OR 1.43, 1.11–2.06) and PPT ratio per 0.1 increase (OR 0.69, 0.50–0.92) were associated with the outcome; under the worst-pain definition only baseline NRS remained associated (OR 1.18, 1.02–1.39), with attenuation of both age (OR 1.34, 0.98–1.86) and PPT ratio (OR 0.86, 0.68–1.08). Apparent areas under the receiver operating characteristic curve (AUCs) were 0.863 and 0.740, respectively; the optimism-corrected AUC for the average-pain model was 0.831. The PPT ratio was strongly correlated with bedside allodynia (Spearman ρ = −0.82). **Conclusions**: PPT asymmetry was associated with one-month pain persistence under one of two outcome definitions but overlapped substantially with clinically assessed allodynia. These exploratory associations require prespecified confirmation before any clinical application.

## 1. Introduction

Herpes zoster (HZ) results from reactivation of latent varicella-zoster virus in sensory ganglia and is one of the most common neurocutaneous conditions in adults, with incidence rising markedly with age [1,2,3]. The acute episode is frequently accompanied by neuritic pain, dysesthesia, pruritus, and stimulus-evoked pain. Although pain improves in most patients as the cutaneous eruption resolves, a clinically important subgroup experiences prolonged zoster-associated pain that substantially interferes with sleep, mood, mobility, work, and other activities of daily living [4,5]. Postherpetic neuralgia (PHN) is generally reserved for dermatomal pain persisting at least 90 days after rash onset; pain present at approximately one month is therefore more appropriately described as subacute or persistent zoster-associated pain rather than established PHN [4,6,7]. In most patients, acute pain declines substantially as the eruption crusts and heals over roughly two to four weeks, but a minority follow a protracted course; reported estimates of the proportion progressing to PHN vary widely, from below 10% to around 30%, depending on age, the pain threshold applied, and the interval used to define persistence [2,4,7]. This heterogeneity in both trajectory and definition is the reason the present study reports an intermediate, approximately one-month time point under two explicit pain-intensity definitions rather than a single dichotomous PHN label.

Older age and greater acute pain severity are among the most consistently reported risk factors for prolonged pain after HZ. Prodromal pain, greater rash severity and ophthalmic involvement have also been associated with subsequent PHN [8,9,10]. A recent meta-analysis of 53 cohort studies likewise identified skin-lesion characteristics, later initiation of treatment, and comorbidities as potential risk factors for PHN [11]. Neuropathic pain characteristics may add information beyond pain intensity alone; prospective observational work has linked neuropathic features during acute HZ with later pain persistence [12], and longitudinal descriptions of the natural history of zoster-associated pain show marked between-patient heterogeneity in resolution trajectories [13]. These findings support multidimensional early assessment rather than reliance on age or a single pain score. Because zoster vaccination substantially reduces HZ incidence [14,15], and efficacy against PHN has also been demonstrated in adults aged 70 years or older [14], identifying patients at risk of a protracted course after an incident episode remains complementary to, rather than a substitute for, primary prevention.

Acute HZ also produces heterogeneous somatosensory abnormalities, including mechanical allodynia, hyperalgesia, thermal abnormalities, and sensory loss. Longitudinal studies show that sensory changes may persist or evolve independently of spontaneous pain [16]. Kramer and colleagues used comprehensive quantitative sensory testing (QST) in acute HZ and found abnormalities not only at affected sites but also at contralateral control sites; selected sensory features were associated with PHN at follow-up [17]. Earlier serial QST work, however, did not reliably identify which patients would later develop PHN [18]. Standardized QST protocols have established that somatosensory profiles differ systematically across neuropathic pain syndromes, with dynamic mechanical allodynia being particularly characteristic of PHN [19,20]. Thus, the prognostic value of somatosensory testing remains unsettled, and full QST batteries may be difficult to implement in routine clinics.

A brief pressure-pain assessment may offer a pragmatic alternative. Notably, in the extension of the German Research Network on Neuropathic Pain (DFNS) reference dataset to the trunk, left–right differences were 1.5–2.3 times more sensitive than absolute reference values for mechanical detection and pain thresholds, and PPT was the single most sensitive parameter, with side-to-side differences exceeding 35% already considered abnormal [21]. Affected-to-contralateral PPT asymmetry can be obtained in minutes and may capture a combination of local mechanical hypersensitivity and broader nociceptive processing. The present study therefore evaluated whether baseline clinical factors and a simple PPT ratio were associated with persistent pain approximately one month after acute HZ. Secondary objectives were to characterize changes in pain, neuropathic features, psychological symptoms, and sleep, and to describe pain-related functional interference at follow-up.

## 2. Materials and Methods

### 2.1. Study Design and Setting

This prospective multicenter observational cohort study was conducted at three tertiary hospitals in Türkiye: University of Health Sciences, Gazi Yaşargil Training and Research Hospital, Diyarbakır (the coordinating center; Center 1); Şanlıurfa Training and Research Hospital, Şanlıurfa (Center 2); and University of Health Sciences, Şanlıurfa Mehmet Akif İnan Training and Research Hospital, Şanlıurfa (Center 3). Participants underwent face-to-face assessment during acute HZ and were scheduled for a second assessment approximately one month after rash onset. Baseline assessment occurred 1–7 days after rash onset (median 3 days), and follow-up visits were completed 23–37 days after rash onset (mean 29.8 ± 3.3 days). Participants were recruited consecutively between 24 June and 11 July 2026 after ethics approval and required site-specific institutional permissions were obtained; one-month follow-up assessments were completed by 10 August 2026. Recruitment occurred across high-volume tertiary outpatient clinics serving large catchment populations, which permitted consecutive enrollment of a large number of incident HZ presentations within a short calendar window.

### 2.2. Participants

Consecutive adults aged 18 years or older with acute HZ, diagnosed clinically by a dermatologist on the basis of a dermatomal vesicular eruption, were eligible if they were assessed within 7 days of rash onset, provided written informed consent, and were considered able to attend the scheduled follow-up assessments. The diagnosis was clinical; virological confirmation was not performed.

Patients were not enrolled if they had pre-existing chronic pain in the same dermatome before the HZ episode, an HZ diagnosis that could not be clinically confirmed, presentation more than 7 days after rash onset, advanced cognitive impairment or communication difficulty precluding reliable pain and sensory assessment, severe peripheral neuropathy or central nervous system disease materially affecting sensory assessment in the same region, a history of postherpetic neuralgia in the same dermatome, another active dermatological condition causing pain or sensory disturbance at the assessment site, any clinical condition preventing completion of the study assessments, or if written informed consent was not given.

Diabetes mellitus, malignancy, immunosuppression, and other chronic conditions were not exclusion criteria in themselves and were recorded as candidate clinical variables; patients in whom advanced neuropathy prevented reliable assessment of HZ-related sensory findings could nonetheless be excluded.

Participants could withdraw at any time without giving a reason and without affecting their treatment, follow-up, or right to care. Participants were considered withdrawn if they did not attend follow-up assessments, could not be contacted by telephone or other permitted means, stated that they no longer wished to participate, withdrew consent, or if the study assessments could not be completed. Participants were removed from the dataset if eligibility criteria were subsequently found not to have been met, if the HZ diagnosis changed, if records were found to be duplicated, or if data reliability was compromised; the reason for removal was recorded in each case.

### 2.3. Assessors, Training and Standardization

Demographic, clinical, and dermatological variables were assessed in the dermatology outpatient clinics of the participating centers by dermatologists who were members of the research team. The HZ diagnosis, dermatomal distribution, lesion characteristics, rash severity and cutaneous healing status were recorded by a dermatologist.

Neurological and sensory assessments were performed by dermatologists or neurologists trained in the study protocol, using a standardized assessment form; participants judged to require it on clinical grounds were additionally evaluated by a neurologist. Because the participating hospitals are in different cities, assessments were carried out by the study investigators based at each center rather than a single assessor visiting all sites; diagnosis, baseline assessment and follow-up assessment for any given participant were performed at the center at which that participant was enrolled. Administration of the DN4 and assessment of mechanical allodynia, light touch, monofilament, pinprick, and warm–cold thermal testing followed the same protocol at every center.

Before the study began, investigators at each center received standardized training in the assessment methods, the order of testing, measurement of the allodynic area, data recording, and follow-up timing. Investigators used a common study operations manual and a standard follow-up form to reduce between-center variation in administration. Data were entered at each center onto a coded standard case record form; the coded datasets were pooled under the supervision of the principal investigator, and data checking and statistical analysis were performed on the combined dataset.

Laboratory values were obtained from tests performed in the biochemistry, hematology, and microbiology laboratories of the respective hospitals during routine clinical care. Laboratory testing was requested according to the treating physician’s clinical judgment rather than according to the study protocol; laboratory data were therefore selectively available and were not used in the core analysis.

The HZ diagnosis, dermatological examination, identification of the involved dermatome, grading of lesion severity, enquiry about pain intensity, assessment of neuropathic pain features, recording of treatments and clinical follow-up all form part of the routine clinical management of patients with HZ. Light touch, pinprick, warm–cold discrimination, monofilament examination and assessment of overt sensory loss are likewise routine neurological examination techniques applied where clinically indicated, and the DN4 is a standard clinical instrument for characterizing neuropathic pain. The study-specific components were the performance of the sensory examination in the same order and with the same standardized form in every participant, standardized pressure algometry, delineation and measurement of the boundaries of the mechanical allodynic area, repetition of the DN4 at the predefined follow-up time points, and systematic transfer of clinical data to the study case record form. No study-specific drug administration, treatment modification, blood sampling, biological specimen collection, genetic analysis, imaging, skin biopsy, or invasive procedure was undertaken.

### 2.4. Baseline Clinical Assessment

Baseline variables included age, sex, body mass index, smoking and alcohol status, education, employment, diabetes mellitus, hypertension, renal and hepatic disease, malignancy, hematologic disease, human immunodeficiency virus (HIV) infection, autoimmune/rheumatologic disease, transplantation, chronic neuropathy, fibromyalgia, depression/anxiety, sleep disorder, immunosuppression, previous systemic corticosteroid or biologic treatment, prior HZ, zoster vaccination and family history of PHN. HZ-specific variables included prodromal pain, time from rash onset to first assessment, side and dermatome, number of involved dermatomes, disseminated or mucosal involvement, ophthalmic or otic involvement, motor deficit, lesion burden, affected area, and global rash severity.

Antiviral treatment, treatment timing, and initiation within 72 h were recorded, consistent with guideline recommendations to start antiviral therapy as early as possible after rash onset [6]. Available laboratory measurements included complete blood count, C-reactive protein, fasting glucose, glycated hemoglobin, creatinine/estimated glomerular filtration rate, transaminases, vitamin B12 and thyroid-stimulating hormone.

### 2.5. Pain and Somatosensory Assessment

Pain intensity was assessed on a 0–10 numerical rating scale (NRS) for current pain, average pain during the preceding 24 h and worst pain during the preceding 24 h. Neuropathic pain features were assessed using the Douleur Neuropathique 4 (DN4) questionnaire; a score ≥4 was considered a positive screen for neuropathic pain [22]. The DN4 has undergone formal linguistic validation for international use and has been validated in Turkish, in which it shows high reliability and sensitivity for identifying neuropathic pain [23,24]. Additional symptoms included nocturnal pain, burning, electric-shock sensations, stabbing/needling sensations, numbness, and pruritus.

Bedside sensory examination recorded allodynia, pinprick hyperalgesia, reduced light-touch sensation, reduced pinprick sensation, cold and warm sensory abnormalities, and perceived side-to-side sensory difference. Pressure-pain threshold (PPT) was assessed using a computerized digital pressure algometer (AlgoMed, Medoc Ltd., Ramat Yishai, Israel) equipped with a 1.0 cm^2^ rubber-tipped probe. Three devices of the same make and model were used, one at each center, and each device underwent the manufacturer-specified calibration procedure before the study began; no single device was shared between centers. Measurements were not performed directly over vesicles, erosions, crusts, or visibly inflamed skin. The probe was positioned on intact skin approximately 2 cm from the nearest lesion border; when this was not anatomically feasible, we used a standardized 5 cm perilesional site. The 2 cm site was used in 88 of 117 participants (75.2%) and the 5 cm site in 29 (24.8%). The distance used was recorded on the paper case record form but was not transferred to the electronic analytic dataset at the participant level, so measurements could not be linked to individual records and a distance-stratified sensitivity analysis was not possible. Anatomically corresponding contralateral intact skin served as the comparison site. The probe was applied perpendicular to the skin, and pressure was increased at a constant rate of 30 kPa/s. Three measurements were obtained at each site, with a 30 s inter-trial interval to minimize temporal summation. Participants were instructed to press a response unit at the exact moment the sensation of pressure first became painful, which immediately terminated the application; an upper safety limit of 1000 kPa was enforced. The mean of the three trials was defined as the PPT for each evaluated site; only this mean was retained in the analytic dataset, and individual trial values were not stored. Baseline PPT measurements at both the affected and the contralateral site were available for all 117 enrolled participants, so no participant was excluded from the ratio for missing index-test data. The interval between the last analgesic dose and algometry was recorded for every participant (median 5.8 h, interquartile range [IQR] 3.2–9.6; range 0.8–21.1 h); measurements were not scheduled at a fixed interval after dosing, and this interval was therefore examined as a covariate rather than controlled by design. Finally, an affected-to-contralateral PPT ratio was calculated, where lower ratios indicated greater relative pressure sensitivity at the affected site. Pressure algometry was performed by protocol-trained dermatologists or neurologists as described above, and the sequence of sensory testing was standardized across centers using the common study operations manual.

### 2.6. Psychological Symptoms, Sleep and Functional Interference

Anxiety and depressive symptoms were assessed with the Hospital Anxiety and Depression Scale (HADS), and sleep quality with the Pittsburgh Sleep Quality Index (PSQI), at baseline and follow-up [25,26,27,28]. At follow-up, pain-related interference was assessed across seven Brief Pain Inventory (BPI) domains: general activity, mood, walking ability, work, relations/social activity, sleep and enjoyment of life [29,30].

### 2.7. Outcomes

Persistent zoster-associated pain at approximately one month was operationalized in two ways, both applied to the 24 h preceding the follow-up assessment: average NRS pain ≥ 3 and worst NRS pain ≥ 3. The average-pain definition was adopted because average intensity is the summary most directly comparable with the baseline measure used in the models and is less influenced by isolated pain peaks; the worst-pain definition is the convention validated against functional impact in the zoster-adapted Brief Pain Inventory [29]. No hierarchy between these two definitions was documented in a dated protocol or statistical analysis plan before the data were locked. The designation of the average-pain definition as the principal analysis is therefore post hoc, and both definitions are reported with equal prominence throughout, including in the Abstract. The term PHN is not applied to either definition because follow-up preceded the conventional ≥90-day interval [4,6]. Additional outcomes included any ongoing pain, DN4 ≥ 4, allodynia, changes in NRS/DN4/HADS/PSQI scores and BPI functional interference.

### 2.8. Statistical Analysis

Continuous variables were summarized as mean ± standard deviation (SD) or median (IQR), as appropriate, and categorical variables as *n* (%). Paired baseline–follow-up comparisons used the Wilcoxon signed-rank test. For persistent-pain group comparisons, continuous baseline variables were compared using Welch’s *t* test and categorical variables using two-sided Fisher exact tests; BPI interference scores were compared using Mann–Whitney U tests because of their strongly skewed, zero-inflated distribution. We compared the eight participants without completed follow-up with retained participants on key baseline characteristics as an attrition analysis. Abbreviations used throughout are defined at first mention.

No formal sample-size calculation was performed. The sample was fixed by the recruitment window, and the 25 events observed under the average-pain definition fall far below the number required to develop a stable multivariable prediction model with acceptable optimism [31]. The multivariable analysis is therefore exploratory throughout: it estimates adjusted associations, quantifies their instability, and is not a prediction-model development study. It is reported as such, and no risk score, nomogram or decision threshold is derived.

Six candidate predictors were initially examined in univariable analyses: age, baseline average 24 h NRS, affected-to-contralateral PPT ratio, baseline allodynia, prodromal pain, and severe rash. After reviewing these results, age (per 10-year increase), baseline average 24 h NRS (per 1-point increase), and PPT ratio (per 0.1 increase) were included in the adjusted model. Because this selection was based on the observed data rather than a prespecified model, the multivariable analysis was considered exploratory. Firth bias-reduced logistic regression was used to limit small-sample bias [32,33]. Model discrimination was assessed using the area under the receiver operating characteristic curve (AUC), while the calibration intercept, calibration slope, and Brier score were reported as apparent measures of model performance.

To account for the data-driven selection process, bootstrap internal validation repeated the full modelling procedure within each of 400 resamples. In each resample, all six candidate predictors were screened univariably, the three predictors with the smallest *p* values were selected, and the model was refitted and evaluated in the original cohort. The resulting optimism-corrected AUC therefore incorporates uncertainty arising from predictor selection. For comparison, an additional bootstrap analysis was performed with the same three predictors held fixed across resamples. Selection frequencies for each candidate predictor were also recorded as an indication of model stability. The apparent AUC of the age-plus-NRS model was further compared with that of the model including the PPT ratio using paired bootstrap resampling.

Several additional exploratory analyses were performed to assess the robustness of the findings. These included center-adjusted Firth regression, use of the alternative worst-pain outcome definition, additional adjustment for the interval since the last analgesic dose, replacement of the PPT ratio with affected-site PPT, contralateral PPT, or the absolute side-to-side PPT difference, and a model containing both the PPT ratio and baseline allodynia. Associations between baseline NRS and PPT ratio, as well as between PPT ratio and allodynia, were examined using Pearson and Spearman correlation coefficients. Variance inflation factors (VIFs) were calculated for the principal predictors to assess multicollinearity.

All tests were two-sided, with *p* < 0.05 considered statistically significant. Descriptive analyses, between-group comparisons, and paired nonparametric tests were performed using IBM SPSS Statistics version 28.0 (IBM Corp., Armonk, NY, USA). Advanced analyses were conducted in Python 3.13.5 using NumPy 2.3.5, SciPy 1.17.0, pandas 2.2.3, and statsmodels 0.14.6. Statsmodels was used for calibration analyses and VIF calculations. Firth logistic regression was implemented using the Jeffreys-prior adjusted-score formulation described in the methodological literature [32,33]. The implementation was cross-checked against direct numerical maximization of the Jeffreys-penalized likelihood, with coefficient estimates agreeing to at least three decimal places. We used a fixed random seed (20260810) for all bootstrap analyses, and archived the complete analysis code and console output with the study files. Reporting followed the STROBE recommendations for observational studies [34]. Because the aim was to explore adjusted associations rather than to develop a clinical prediction model, prediction-model reporting guidance was not applied.

Before the final analysis, the dataset underwent quality-control checks. Non-informative “unknown” responses for zoster vaccination status (*n* = 28) and family history of PHN (*n* = 33) were treated as missing rather than as valid clinical categories. Nine recorded healing times exceeded the documented follow-up interval and were therefore considered implausible and set to missing for analyses involving healing time. These corrections did not affect either outcome definition, baseline NRS, PPT ratio, the 109-participant follow-up denominator, or the 25 events identified using the average-pain definition.

## 3. Results

### 3.1. Cohort Characteristics and Follow-Up

We assessed 120 patients with clinically diagnosed acute HZ for eligibility. Three declined participation, and 117 provided written informed consent and were enrolled (Figure 1). Of these, 54 were recruited at Center 1, 35 at Center 2, and 28 at Center 3. The mean age of the cohort was 58.7 ± 14.7 years, and 71 participants (60.7%) were women. Diabetes mellitus was present in 22 participants (18.8%), hypertension in 39 (33.3%), and immunosuppression in 19 (16.2%). Prodromal pain was reported by 70 participants (59.8%). Thoracic involvement was the most common dermatomal distribution (68/117, 58.1%), followed by trigeminal involvement (24/117, 20.5%). Rash severity was classified as mild in 49 participants (41.9%), moderate in 47 (40.2%), and severe in 21 (17.9%). The baseline characteristics are summarized in Table 1.

Antiviral treatment was initiated in 111 of 117 participants (94.9%), and 64 of these 111 participants (57.7%) started treatment within 72 h of rash onset. Valaciclovir was used in 70 participants, aciclovir in 26, famciclovir in 11, and brivudine in 4. Systemic corticosteroids were co-administered at baseline in 15 participants (12.8%). All participants were receiving a simple analgesic at baseline: paracetamol in 39 (33.3%), diclofenac in 31 (26.5%), naproxen in 27 (23.1%), and flurbiprofen in 20 (17.1%). None was receiving an opioid, anticonvulsant, antidepressant, or topical anesthetic at the baseline assessment.

The mean baseline 24 h NRS score was 5.74 ± 3.19. A DN4 score ≥ 4 was present in 58 participants (49.6%), while allodynia was identified in 51 (43.6%). Mean PPT was 260.0 ± 89.5 kPa at the affected site and 307.4 ± 74.4 kPa at the contralateral site, giving a mean affected-to-contralateral PPT ratio of 0.842 ± 0.185.

One-month follow-up was completed by 109 of 117 participants (93.2%); eight participants (6.8%) were lost to follow-up. No statistically significant differences were observed between retained and lost participants with respect to age, sex, baseline average NRS, PPT ratio, allodynia, prodromal pain, or severe rash. Among participants who completed follow-up and received antiviral treatment, 58 of 103 (56.3%) had started therapy within 72 h, compared with 6 of 8 participants lost to follow-up (75.0%; Fisher exact *p* = 0.290).

### 3.2. One-Month Clinical Course and Functional Burden

Among 109 participants with recorded follow-up at a mean of 29.8 ± 3.3 days after rash onset (range 23–37 days), 45 (41.3%) reported some ongoing pain. Average 24 h NRS ≥ 3 was present in 25/109 (22.9%), of whom 17 had average NRS 3–6 and eight had average NRS ≥ 7; worst 24 h NRS ≥ 3 was present in 42/109 (38.5%). Neuropathic pain treatment had been started by the follow-up visit in 23/109 participants (21.1%): gabapentin in 6, pregabalin in 6, amitriptyline in 5, duloxetine in 4 and topical lidocaine in 2. Seventeen of these 23 met the average-pain outcome, so this treatment was initiated in response to persisting pain and lies on the pathway between the acute episode and the outcome; it is reported descriptively and was not entered into any model. DN4 remained ≥4 in 12/109 (11.0%), and allodynia was present in 12/109 (11.0%).

Average 24 h NRS decreased from 5.73 ± 3.23 to 1.52 ± 2.34 (Wilcoxon *p* < 0.001), and DN4 from 3.61 ± 1.71 to 2.04 ± 1.33 (*p* < 0.001). HADS anxiety decreased by 0.75 points, HADS depression by 0.61 points and PSQI by 1.00 point (all *p* < 0.001). These psychological and sleep changes were statistically significant but small in absolute magnitude and should not be interpreted as evidence of a large clinically meaningful improvement.

Functional interference was markedly greater among participants meeting the persistent-pain outcome. Median overall BPI interference across the seven domains was 3.14 (IQR 2.57–4.43) in the persistent-pain group versus 0 (IQR 0–0) in those without persistent pain (*p* < 0.001; corresponding means 3.46 and 0.28). Sleep showed the largest between-group contrast (median 4, IQR 4–6, versus 0, IQR 0–0), and all seven individual BPI domains differed at *p* < 0.001 (Table 2).

### 3.3. Baseline Factors Associated with Persistent Pain

Participants with persistent pain were older than those without persistent pain (68.0 ± 11.7 vs. 56.4 ± 13.7 years; *p* < 0.001), had greater baseline average 24 h pain intensity (8.08 ± 2.08 vs. 5.04 ± 3.19; *p* < 0.001), and had a lower affected-to-contralateral PPT ratio (0.746 ± 0.164 vs. 0.876 ± 0.181; *p* = 0.001) (Table 3). Among participants with baseline allodynia, 16/47 (34.0%) developed persistent pain compared with 9/62 (14.5%) without allodynia. Corresponding rates were 20/66 (30.3%) versus 5/43 (11.6%) for prodromal pain, and 8/19 (42.1%) versus 17/90 (18.9%) for severe versus non-severe rash. Baseline DN4 ≥ 4 was not significantly associated with either outcome definition. The PPT ratio was closely related to bedside allodynia: the mean ratio was 0.674 ± 0.118 in the 47 participants with baseline allodynia and 0.976 ± 0.100 in the 62 without (Welch *p* < 0.001; Spearman ρ = −0.82, *p* < 0.001).

Among antiviral-treated participants with follow-up, treatment within 72 h occurred in 14/23 (60.9%) with persistent pain and 44/80 (55.0%) without persistent pain (Fisher exact *p* = 0.643). This analysis uses treated participants as the denominator and should not be interpreted causally because treatment timing was not randomized.

In univariable Firth models, persistent pain was associated with age (odds ratio [OR] 1.96 per 10 years, 95% confidence interval [CI] 1.32–2.90), baseline average NRS (OR 1.65 per point, 1.24–2.20), allodynia (OR 2.95, 1.18–7.40), prodromal pain (OR 3.09, 1.09–8.77), severe rash (OR 3.10, 1.09–8.87) and lower PPT ratio (OR 0.68 per 0.1 increase, 0.53–0.88). In the three-predictor penalized model applied to the average-pain definition, age (adjusted OR 1.60 per 10 years, 1.03–2.59; *p* = 0.036), baseline average NRS (1.43 per point, 1.11–2.06; *p* = 0.003) and PPT ratio (0.69 per 0.1 increase, 0.50–0.92; *p* = 0.010) were associated with the outcome. Applied to the worst-pain definition, only baseline NRS retained an association (1.18 per point, 1.02–1.39; *p* = 0.025); age (1.34 per 10 years, 0.98–1.86; *p* = 0.066) and the PPT ratio (0.86 per 0.1 increase, 0.68–1.08; *p* = 0.205) were attenuated (Table 4).

### 3.4. Exploratory Model Performance, Alternative Metrics, and Sensitivity Analyses

Under the average-pain definition, the three-predictor model had an apparent AUC of 0.863; under the worst-pain definition, the corresponding value was 0.740. Apparent calibration intercept was 0.019, calibration slope 1.10, and Brier score 0.119 for the average-pain model. The age-plus-NRS model had an apparent AUC of 0.820; adding the PPT ratio increased AUC by 0.044, but the paired bootstrap 95% CI for this increment included zero (−0.007 to 0.102).

When bootstrap internal validation repeated the univariable screening and three-variable selection within each resample, estimated optimism was 0.032, and the optimism-corrected AUC was 0.831. Holding the same three predictors fixed across resamples gave a smaller optimism of 0.021 and a corrected AUC of 0.842; the difference of 0.011 is the discrimination attributable to the selection step itself. Across resamples, baseline NRS was selected in 99.5%, age in 85.8% and the PPT ratio in 72.5%, whereas prodromal pain (18.5%), allodynia (14.2%) and severe rash (9.5%) were selected infrequently. The PPT ratio therefore enters roughly three of every four models built from resampled versions of these data, which represents moderate rather than strong stability (Table 5).

Substituting the PPT ratio with simpler measurements showed that the within-person contrast, rather than single-site value, carried the signal (Table 6). Contralateral PPT alone was entirely uninformative (OR 1.06 per 50 kPa, 95% CI 0.76–1.49) and affected-site PPT alone did not reach significance (OR 0.82 per 50 kPa, 0.61–1.09). The absolute side-to-side difference in kPa performed at least as well as the ratio (OR 1.91 per 50 kPa, 1.18–3.31; apparent AUC 0.871 versus 0.863 for the ratio), indicating that the ratio form is not itself necessary.

When baseline allodynia was entered alongside the PPT ratio, neither variable retained an independent association (PPT ratio OR 0.63 per 0.1 increase, 95% CI 0.38–1.02, *p* = 0.060; allodynia OR 0.68, 0.11–4.03, *p* = 0.668) and apparent discrimination was essentially unchanged (AUC 0.866 versus 0.863). Given the correlation of ρ = −0.82 between the two variables, these estimates should be read as evidence that the ratio and bedside allodynia capture largely the same construct rather than as evidence that either is independently unimportant.

Adjusting additionally for the interval since the last analgesic dose left the estimates essentially unchanged (PPT ratio OR 0.686 per 0.1 increase, 95% CI 0.50–0.92; interval OR 0.95 per hour, 0.84–1.07). The interval was unrelated to the PPT ratio (ρ = −0.04, *p* = 0.66) and did not differ between outcome groups (7.0 versus 6.8 h, *p* = 0.64).

Outcome rates differed across centers: 13/50 (26.0%) in Center 1, 3/31 (9.7%) in Center 2 and 9/28 (32.1%) in Center 3 among participants with follow-up. In a center-adjusted Firth model, baseline NRS (OR 1.40 per point, 95% CI 1.05–1.86) and PPT ratio (OR 0.68 per 0.1 increase, 0.50–0.93) remained associated with persistent pain; the age estimate was attenuated (OR 1.54 per 10 years, 0.96–2.47). Baseline characteristics by center are given in Supplementary Table S1: participants at Center 2 were on average 13 years younger than those at Center 3 (52.7 versus 65.6 years, *p* = 0.001) and reported prodromal pain less often (41.9% versus 71.4%, *p* = 0.039), whereas the PPT measurements themselves—ratio, affected-site and contralateral values—did not differ across centers (*p* = 0.45, 0.35 and 0.36), arguing that the between-center variation in outcome reflects case mix rather than differences in algometry.

Baseline average NRS and PPT ratio showed modest inverse correlation (Pearson *r* = −0.287; Spearman ρ = −0.236), and VIFs for age, NRS and PPT ratio were 1.12, 1.22 and 1.09, respectively, arguing against problematic multicollinearity among the modelled variables.

## 4. Discussion

In this multicenter cohort, 22.9% of participants with completed follow-up met the average-pain definition of persistent zoster-associated pain approximately one month after rash onset, and 38.5% met the worst-pain definition. Older age and greater acute pain intensity were associated with this early outcome, consistent with established HZ risk patterns. Baseline allodynia, prodromal pain, and severe rash were also associated with persistent pain in univariable analyses. The principal new observation was that a lower affected-to-contralateral PPT ratio remained associated with the average-pain outcome after adjustment for age and average acute pain intensity, and the association persisted after adjustment for coded center.

The clinical associations provide an important validity check. Prospective cohorts and the systematic review by Forbes and colleagues consistently identify older age, severe acute pain, prodromal pain, and severe rash as risk factors for subsequent PHN [8,9,10]. Our one-month endpoint is earlier than PHN and should not be equated with chronic neuropathic pain, but the direction of these associations supports a biologically coherent transition from the acute episode to persistent pain.

Interpretation of the PPT ratio requires particular care in light of prior QST literature. Kramer et al. assessed 74 patients with acute HZ using a comprehensive sensory battery at affected and distant contralateral control sites and compared them with healthy controls [17]. The affected site showed multiple gain- and loss-of-function abnormalities, including pressure hyperalgesia; importantly, the contralateral control site also showed thermal and vibratory hypesthesia, dynamic mechanical allodynia and pressure hyperalgesia. PHN at six months was associated with dynamic mechanical allodynia and vibratory hypesthesia at the control site. This is consistent with a wider literature indicating that the unaffected side in unilateral neuropathic conditions is not necessarily physiologically normal: skin biopsy work has demonstrated bilateral loss of epidermal nociceptors in unilateral PHN [35], and standardized QST in unilateral neuropathic pain has shown sensory abnormalities on the contralateral side, albeit to a lesser degree than on the painful side [36]. If bilateral or generalized changes affect both sides similarly, a side-to-side ratio could attenuate contrast toward unity, but the direction and magnitude of this bias cannot be established from the present data. We therefore interpret the ratio as within-person asymmetry rather than as a pure measure of local hyperalgesia.

At the same time, the DFNS trunk reference dataset supports the measurement strategy used here: for mechanical detection and pain thresholds on the trunk, left–right differences were substantially more sensitive than absolute reference values, and PPT was the most sensitive individual parameter, with side-to-side differences greater than 35% falling outside the normal range [21]. In our cohort, the mean affected-to-contralateral PPT ratio in participants who developed persistent pain corresponded to approximately 25% asymmetry, which is within the range that would not be classified as abnormal by that criterion; the association we observed is therefore a graded, group-level one rather than evidence that individual participants crossed an established abnormality threshold. In addition, our 30-kPa/s pressure ramp differs from the 50-kPa/s rate specified in the standard DFNS pressure-pain protocol [19], which should be considered when comparing absolute PPT values with DFNS-based QST studies such as Kramer et al. [17] and Pfau et al. [21].

The most consequential limitation of the index test emerged from an analysis requested during peer review. The PPT ratio proved to be closely correlated with clinically assessed dynamic mechanical allodynia (Spearman ρ = −0.82): the mean ratio was 0.674 among participants with allodynia and 0.976 among those without. When both variables were entered into the same model, neither retained an independent association, and apparent discrimination increased only from 0.863 to 0.866. The most parsimonious reading is that perilesional pressure applied 2 cm from the lesion border frequently falls within the allodynic field, so that the algometer registers dynamic mechanical allodynia at least as much as pressure hyperalgesia. The PPT ratio is therefore better described as a continuous, quantitative index of perilesional mechanical hypersensitivity that overlaps substantially with a finding already obtainable at the bedside, rather than as a measurement that adds a distinct dimension to clinical examination. Its potential value lies in graduation—a continuous scale in place of a present-or-absent judgement, with the reproducibility that instrumented measurement affords—and that potential value has not been demonstrated here.

Comparison with simpler measurements is similarly sobering in one respect and reassuring in another. Neither single-site value carried the association: the contralateral threshold alone was entirely uninformative and the affected threshold alone did not reach significance, which supports the premise that the within-person contrast, and not the absolute threshold, is what matters. However, the absolute side-to-side difference in kPa performed at least as well as the ratio, so the ratio form confers no demonstrable advantage and future work need not adopt it. We report the ratio because it was the measurement specified in our assessment protocol, not because the data identify it as the preferable metric.

Our findings should also be viewed alongside negative sensory-prediction literature. Haanpää, Laippala and Nurmikko performed serial thermal and tactile QST in 97 immunocompetent patients with acute HZ and found that early threshold abnormalities reflected impaired sensory function but did not identify which patients later developed PHN [18]. This distinction is important: our approach does not establish that PPT is a universal sensory biomarker. Its potential contribution is pragmatic—a brief pressure algometry measure that can be obtained within minutes rather than a full DFNS-style battery, which is relevant given that comprehensive protocols require specialized equipment and substantial assessment time [19,20]. Even so, the current signal requires replication.

A second interpretive issue is tissue inflammation. Although PPT measurements were deliberately obtained on intact perilesional skin rather than directly over vesicles, erosions, crusts, or visibly inflamed skin, subclinical or perifocal inflammatory changes could still influence mechanical pain sensitivity. The standardized 2 cm perilesional site, with use of a 5 cm site when the 2 cm location was not anatomically feasible, was intended to reduce direct lesion-related measurement bias. Nevertheless, the present observational design cannot fully separate peripheral inflammatory hyperalgesia from neural injury or altered nociceptive processing; the PPT ratio should therefore be interpreted as a pragmatic within-person measure of sensory asymmetry rather than a mechanism-specific biomarker.

Model-performance analyses reinforce the need for restraint, and the framing of this analysis warrants an explicit statement. The three variables carried into the adjusted model were chosen after the univariable results had been inspected; the selection was data-driven and was not specified in advance in any dated document. Bootstrap internal validation that repeats this selection within every resample gives an optimism-corrected AUC of 0.831, compared with 0.842 when the same three predictors are held fixed, so the selection step costs approximately 0.011 of discrimination. The selection frequencies are a more informative summary than either figure: baseline pain intensity is selected in almost every resample and age in most, whereas the PPT ratio is selected in roughly three of four, which is moderate stability. The apparent calibration slope of 1.10 is consistent with the mild shrinkage imposed by Firth penalization rather than with overfitting, and calibration and Brier values are reported as apparent quantities that were not themselves internally validated. Taken together with a ΔAUC of 0.044 whose bootstrap interval crosses zero, and with the attenuation of the PPT association under the worst-pain definition, these analyses do not support a claim of incremental predictive value. They support a description of association, examined under one of two reasonable outcome definitions, in a sample far smaller than would be needed to develop a stable prediction model [31]. For this reason, we present no risk score, nomogram, or decision threshold, and we have not adopted prediction-model reporting guidance, which would imply a study design this analysis does not have.

Center-specific outcome rates ranged from 9.7% to 32.1%. The center-wise comparison in Supplementary Table S1 indicates that this reflects case mix rather than measurement: participants at the center with the lowest event rate were on average 13 years younger and reported prodromal pain less often, while the PPT measurements themselves did not differ significantly across centers despite the use of three separate calibrated devices of the same model. The PPT association also remained in a center-adjusted model, although confidence intervals were wide and age was attenuated. Standardization of algometry and sensory examination across sites nonetheless remains essential for external reproducibility.

Pain-related functional interference was substantially greater among participants meeting the persistent-pain definition, with sleep showing the largest absolute burden. In contrast, cohort-level changes in HADS and PSQI were statistically significant but small in absolute magnitude. These findings highlight the distinction between statistical and clinical significance and suggest that persistent pain burden at one month is more clearly expressed in pain-related interference than in large average shifts in psychological symptom scales.

Concomitant analgesic treatment deserves comment because the outcome is pain intensity. Baseline analgesic exposure was unusually homogeneous: every participant was taking either paracetamol or a non-selective non-steroidal anti-inflammatory drug, and none was receiving an opioid, anticonvulsant, antidepressant, or topical anesthetic when algometry was performed. This homogeneity limits the scope for confounding by analgesic class, although it does not exclude dose-related effects, which were not recorded. The interval since the last analgesic dose was recorded for every participant and, when entered as a covariate, left the estimates essentially unchanged. Neuropathic pain treatment had been started in 21.1% of participants by follow-up, overwhelmingly in those with persisting pain; because such treatment is a consequence of the outcome rather than a cause, adjusting for it would introduce collider bias, and it is reported descriptively only. Analgesic use at the follow-up visit was not systematically recorded, and this remains a genuine gap.

The absence of an association between early antiviral timing and the one-month outcome should not be interpreted as evidence against guideline-concordant antiviral treatment [6]. Nearly all participants received antivirals, treatment timing was observational, and confounding by indication is likely. The analysis is therefore descriptive and cannot estimate treatment efficacy. Similarly, the most effective means of reducing the population burden of persistent zoster-associated pain remains prevention of the incident episode through vaccination [14,15].

This study has several limitations. Follow-up at approximately one month precludes assessment of established PHN. The sample size was modest and only 25 events occurred under the average-pain definition, limiting precision even with penalized regression, and it was determined by the recruitment window rather than by a formal calculation [31]. Neither the outcome hierarchy nor the predictor set was prespecified in a dated protocol or statistical analysis plan; both were determined after the data were available, which is why both outcome definitions are reported in full, and the selection step is incorporated into the internal validation. The worst-pain definition weakened the PPT association, and this endpoint dependence is a substantive finding, not a footnote. Contralateral tissue may not be a physiologically normal comparator, as prior data demonstrate bilateral or distant sensory and structural abnormalities in unilateral zoster-related pain [17,35,36]. PPT may also be influenced by local cutaneous inflammation. Center effects, measurement reproducibility and residual confounding remain concerns. Several limitations concern the index test itself. The analytic dataset retained only the mean of the three trials at each site, so intraclass correlation coefficients and within-participant coefficients of variation could not be estimated retrospectively, and formal competency certification of assessors beyond protocol training was not undertaken. For the same reason, although no site-level mean approached the 1000-kPa safety limit (maximum affected-site mean 458 kPa; maximum contralateral-site mean 470 kPa), it cannot be demonstrated that no individual trial did so, and any such ceiling would compress the ratio non-randomly. The 2 cm site was used in 75.2% of participants and the 5 cm site in 24.8%, but the distance was recorded only on the paper case record forms and was not linked to individual electronic records; this is a source of measurement heterogeneity that could not be examined analytically, since a deeper site necessarily samples different tissue and may lie outside a small allodynic field. The findings also do not extend to zoster sine herpete. Our eligibility criteria exclude this atypical presentation and, more fundamentally, the index test itself requires a lesion border from which to measure. The exclusion is not neutral with respect to the outcome studied here: in a comparison of 16 consecutive patients with zoster sine herpete and 16 with typical eruptive zoster, pain severity and duration and analgesic requirement, including opioid use, were greater in the group without rash [37]. The subgroup our design omits may therefore be the one with the most protracted pain, and any estimate of the burden of persistent zoster-associated pain derived from rash-positive cohorts such as ours is likely to be conservative. Whether a contralateral-referenced pressure measurement could be adapted to a dermatome without visible lesions—for instance by referencing the symptomatic dermatome to the corresponding contralateral dermatome rather than to a perilesional site—is an open question that our data cannot address.

Finally, the HZ diagnosis was clinical and was not confirmed virologically, although dermatomal vesicular eruption assessed by a dermatologist is the accepted basis for diagnosis in routine care [6].

Strengths include early assessment during the first week of HZ, a multicenter design, structured multidimensional pain and sensory characterization, high recorded one-month follow-up, explicit attrition analysis, penalized regression with bootstrap internal validation and clinically interpretable functional outcomes. Together, the findings justify a specific next study rather than clinical adoption. A confirmatory cohort should register a dated protocol specifying a single outcome definition and a fixed predictor set before recruitment; retain individual algometry trials so that reliability can be quantified; record the measurement distance and analgesic exposure at the participant level at every visit; measure the algometer response outside as well as inside the allodynic field, so that pressure hyperalgesia can be separated from dynamic mechanical allodynia; and follow participants beyond 90 days so that the endpoint is established PHN rather than an intermediate state. Based on the current evidence, pressure algometry should be regarded as a research measurement in acute zoster. Its plausible near-term role, if confirmed, is to grade the severity of mechanical hypersensitivity already detectable at the bedside—which may prove useful for stratifying participants in trials of early intervention—rather than to identify patients whom clinical examination would otherwise miss.

## 5. Conclusions

Persistent zoster-associated pain approximately one month after rash onset was present in 25/109 participants (22.9%) under an average-pain definition and 42/109 (38.5%) under a worst-pain definition. Older age, greater average acute pain intensity and greater affected-versus-contralateral PPT asymmetry were associated with the average-pain outcome; only acute pain intensity was associated with the worst-pain outcome. The PPT ratio overlapped substantially with clinically assessed allodynia; its incremental contribution to discrimination was uncertain, and the predictors were selected after the univariable results were seen. These findings describe an association between early perilesional mechanical hypersensitivity and short-term pain persistence; they do not establish predictive value, and no clinical use is proposed. Confirmation requires a prespecified protocol, participant-level recording of measurement conditions and follow-up beyond 90 days.

## Figures and Tables

**Figure 1:**
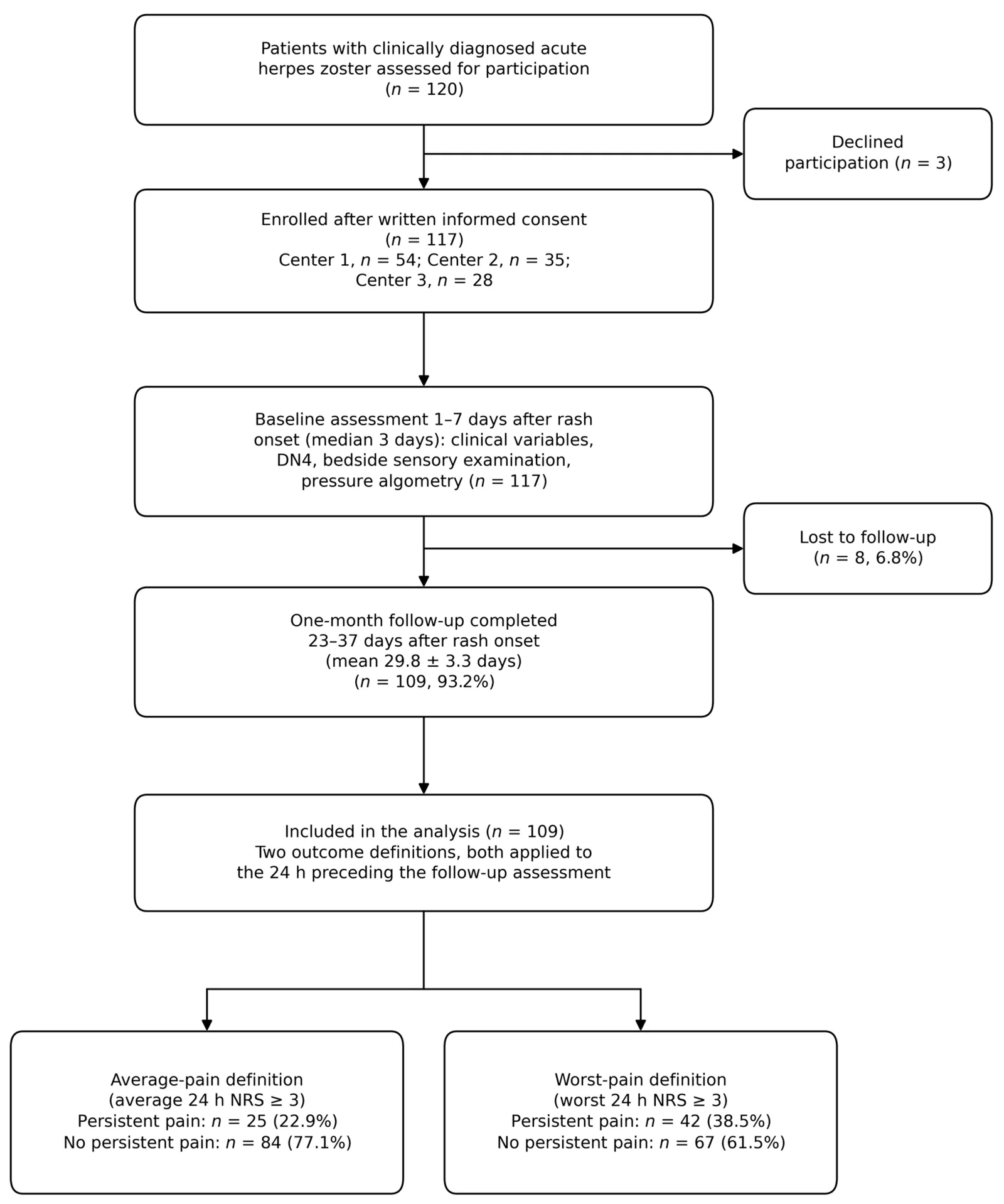
Study flow from screening to the analysis at approximately one month. NRS—numerical rating scale; DN4—Douleur Neuropathique 4.

**Table 1:** Baseline characteristics of the cohort.

Characteristic	Overall (*N* = 117)
Age, years	58.7 ± 14.7
Women	71 (60.7%)
Body mass index, kg/m^2^	27.4 ± 4.6
Diabetes mellitus	22 (18.8%)
Hypertension	39 (33.3%)
Immunosuppression	19 (16.2%)
Prodromal pain	70 (59.8%)
Thoracic dermatome	68 (58.1%)
Trigeminal dermatome	24 (20.5%)
Severe global rash	21 (17.9%)
Baseline average 24 h NRS	5.74 ± 3.19
DN4 ≥ 4	58 (49.6%)
Allodynia	51 (43.6%)
Affected PPT, kPa	260.0 ± 89.5
Contralateral PPT, kPa	307.4 ± 74.4
Affected/contralateral PPT ratio	0.842 ± 0.185
Antiviral treatment initiated	111 (94.9%)
Antiviral ≤ 72 h, among treated	64/111 (57.7%)
HADS anxiety	6.80 ± 3.70
HADS depression	5.82 ± 3.70
PSQI	7.22 ± 3.40

Data are mean ± SD or *n* (%), unless otherwise stated. NRS—numerical rating scale; PPT—pressure-pain threshold; DN4—Douleur Neuropathique 4; HADS—Hospital Anxiety and Depression Scale; PSQI—Pittsburgh Sleep Quality Index.

**Table 2:** Brief Pain Inventory interference at one-month follow-up.

Domain	Persistent Pain (*n* = 25), Median [IQR]	Persistent Pain, Mean ± SD	No Persistent Pain (*n* = 84), Median [IQR]	No Persistent Pain, Mean ± SD	*p*-Value
General activity	4 [3–5]	4.00 ± 1.96	0 [0–0]	0.36 ± 0.87	<0.001
Mood	3 [1–4]	3.16 ± 2.21	0 [0–0]	0.30 ± 0.74	<0.001
Walking ability	2 [2–4]	2.68 ± 1.52	0 [0–0]	0.11 ± 0.35	<0.001
Work	3 [2–4]	3.60 ± 2.24	0 [0–0]	0.37 ± 0.85	<0.001
Relations/social activity	3 [2–4]	2.88 ± 1.48	0 [0–0]	0.31 ± 0.86	<0.001
Sleep	4 [4–6]	4.68 ± 1.57	0 [0–0]	0.23 ± 0.77	<0.001
Enjoyment of life	4 [2–4]	3.20 ± 1.55	0 [0–0]	0.29 ± 0.75	<0.001
Mean across seven domains	3.14 [2.57–4.43]	3.46 ± 1.24	0 [0–0]	0.28 ± 0.55	<0.001

Scores range from 0 (does not interfere) to 10 (completely interferes). Median [IQR] is the primary summary because the distributions are strongly skewed and zero-inflated; mean ± SD is shown alongside for comparability with other zoster cohorts. Between-group *p*-values are from Mann–Whitney U-tests. The interquartile range is 0–0 in the no-persistent-pain group for every individual domain because most of these participants reported no interference; the low but nonzero means reflect a minority with pain below the NRS ≥ 3 threshold and/or residual interference.

**Table 3:** Baseline factors according to one-month persistent pain status.

Variable	Persistent Pain (*n* = 25)	No Persistent Pain (*n* = 84)	*p* Value	Test
Age, years	68.0 ± 11.7	56.4 ± 13.7	<0.001	Welch *t*
Women	17 (68.0%)	50 (59.5%)	0.491	Fisher exact
Body mass index, kg/m^2^	27.8 ± 4.9	27.4 ± 4.5	0.726	Welch *t*
Prodromal pain	20 (80.0%)	46 (54.8%)	0.035	Fisher exact
Severe rash	8 (32.0%)	11 (13.1%)	0.038	Fisher exact
Baseline average 24 h NRS	8.08 ± 2.08	5.04 ± 3.19	<0.001	Welch *t*
DN4 ≥ 4	16 (64.0%)	39 (46.4%)	0.172	Fisher exact
Allodynia	16 (64.0%)	31 (36.9%)	0.022	Fisher exact
Affected/contralateral PPT ratio	0.746 ± 0.164	0.876 ± 0.181	0.001	Welch *t*
HADS anxiety	7.32 ± 3.88	6.61 ± 3.75	0.421	Welch *t*
HADS depression	6.16 ± 3.42	5.83 ± 3.80	0.685	Welch *t*
PSQI	8.48 ± 3.29	7.00 ± 3.32	0.056	Welch *t*
Antiviral ≤ 72 h, among treated	14/23 (60.9%)	44/80 (55.0%)	0.643	Fisher exact

Persistent pain = average 24 h NRS ≥ 3 at follow-up approximately one month after rash onset (range 23–37 days). For antiviral timing, the denominator is participants who received antiviral therapy and completed follow-up. Abbreviations as in Table 1.

**Table 4:** Firth penalized logistic regression under both outcome definitions.

Predictor	Univariable OR (95% CI)	Adjusted OR (95% CI), Average 24 h NRS ≥ 3 (25 Events)	*p*	Adjusted OR (95% CI), Worst 24 h NRS ≥ 3 (42 Events)	*p*
Age, per 10-year increase	1.96 (1.32–2.90)	1.60 (1.03–2.59)	0.036	1.34 (0.98–1.86)	0.066
Baseline average 24 h NRS, per point	1.65 (1.24–2.20)	1.43 (1.11–2.06)	0.003	1.18 (1.02–1.39)	0.025
Affected/contralateral PPT ratio, per 0.1 increase	0.68 (0.53–0.88)	0.69 (0.50–0.92)	0.010	0.86 (0.68–1.08)	0.205
Baseline allodynia	2.95 (1.18–7.40)	—	—	—	—
Prodromal pain	3.09 (1.09–8.77)	—	—	—	—
Severe rash	3.10 (1.09–8.87)	—	—	—	—
DN4 ≥ 4	2.00 (0.80–4.99)	—	—	—	—
Antiviral ≤ 72 h, among treated	1.25 (0.49–3.20)	—	—	—	—
Apparent AUC of the adjusted model	—	0.863	—	0.740	—

Univariable estimates refer to the average-pain definition. The adjusted model contains age, baseline average NRS and PPT ratio; these three variables were selected after inspection of the univariable results, and the selection was therefore data driven. Confidence intervals are profile penalized-likelihood intervals. The antiviral timing analysis is restricted to treated participants. OR—odds ratio; CI—confidence interval; AUC—area under the receiver operating characteristic curve. Other abbreviations as in Table 1.

**Table 5:** Exploratory internal validation, model stability and additional analyses.

Analysis	Result
Apparent AUC, average-pain outcome	0.863
Apparent AUC, worst-pain outcome	0.740
Optimism-corrected AUC, selection repeated within each of 400 resamples	0.831 (optimism 0.032)
Optimism-corrected AUC, three predictors held fixed across resamples	0.842 (optimism 0.021)
Apparent calibration intercept/slope	0.019/1.10
Apparent Brier score	0.119
Increment after adding PPT ratio to age + NRS	ΔAUC 0.044 (bootstrap 95% CI −0.007 to 0.102)
Selection frequency across resamples	NRS 99.5%; age 85.8%; PPT ratio 72.5%; prodromal pain 18.5%; allodynia 14.2%; severe rash 9.5%
Center-adjusted PPT ratio	OR 0.68 per 0.1 increase (95% CI 0.50–0.93)
PPT ratio adjusted additionally for interval since last analgesic	OR 0.69 per 0.1 increase (95% CI 0.50–0.92)
Correlation, PPT ratio with baseline allodynia	Spearman ρ = −0.82 (*p* < 0.001)
Correlation, PPT ratio with baseline NRS	Pearson *r* = −0.287; Spearman ρ = −0.236
VIFs: age/NRS/PPT ratio	1.12/1.22/1.09

Calibration and Brier values are apparent and were not themselves internally validated. VIF—variance inflation factor. Other abbreviations as in Table 1 and Table 4.

**Table 6:** Alternative pressure-pain measurements, each added to a model containing age and baseline average 24 h NRS (average-pain outcome definition).

Measurement	OR (95% CI)	*p* Value	Apparent AUC
Age + NRS only (reference model)	—	—	0.820
Affected/contralateral ratio, per 0.1 increase	0.69 (0.50–0.92)	0.010	0.863
Affected-site PPT alone, per 50 kPa	0.82 (0.61–1.09)	0.180	0.830
Contralateral PPT alone, per 50 kPa	1.06 (0.76–1.49)	0.728	0.820
Absolute side-to-side difference, per 50 kPa	1.91 (1.18–3.31)	0.007	0.871
Ratio and baseline allodynia entered together	ratio 0.63 (0.38–1.02); allodynia 0.68 (0.11–4.03)	0.060; 0.668	0.866

All models are Firth penalized. Abbreviations as in Table 1 and Table 4.

## Data Availability

The de-identified participant-level dataset and the complete analysis code supporting the findings of this study are available from the corresponding author on reasonable request, subject to applicable ethics, institutional, privacy and data-protection requirements.

## Supplementary Materials

The following supporting information can be downloaded at: https://www.mdpi.com/article/10.3390/jcm15197494/s1, Table S1: Baseline characteristics of participants with completed follow-up by recruiting center.
