# Supplementary material for: Clinical and Somatosensory Correlates of Persistent Zoster-Associated Pain at One Month: A Prospective Multicenter Cohort Study
Source: J Clin Med. 2026 Sep 26;15(19):7494. doi: 10.3390/jcm15197494 (PMC13665823; doi:10.3390/jcm15197494)
Supplement: Supplementary file 1 [file jcm-15-07494-s001.zip › jcm-4527689-supplementary.pdf]

Supplementary Materials

Table S1. Baseline characteristics of participants with completed follow-up by recruiting center.

**Variable**	**Center 1 (n = 50)**	**Center 2 (n = 31)**	**Center 3 (n = 28)**	***p* value**
Age, years	59.4 ± 13.2	52.7 ± 13.5	65.6 ± 13.5	0.001
Women	30/50 (60.0%)	18/31 (58.1%)	19/28 (67.9%)	0.712
Days from rash onset to baseline	3.22 ± 1.79	3.16 ± 1.55	3.11 ± 1.62	0.977
Baseline average 24-h NRS	5.92 ± 3.08	4.84 ± 3.28	6.39 ± 3.36	0.117
Baseline DN4	3.62 ± 1.76	3.48 ± 1.65	3.71 ± 1.74	0.957
DN4 ≥4	26/50 (52.0%)	15/31 (48.4%)	14/28 (50.0%)	0.950
Allodynia	19/50 (38.0%)	14/31 (45.2%)	14/28 (50.0%)	0.569
Prodromal pain	33/50 (66.0%)	13/31 (41.9%)	20/28 (71.4%)	0.039
Affected-site PPT, kPa	272.8 ± 86.7	256.6 ± 101.0	247.6 ± 84.8	0.345
Contralateral PPT, kPa	317.9 ± 72.2	298.1 ± 85.8	300.5 ± 71.1	0.363
Affected/contralateral PPT ratio	0.86 ± 0.19	0.85 ± 0.20	0.82 ± 0.15	0.452
Hours since last analgesic dose	7.63 ± 4.47	5.19 ± 4.60	7.16 ± 4.37	0.014
Diabetes mellitus	7/50 (14.0%)	7/31 (22.6%)	7/28 (25.0%)	0.427
Immunosuppression	7/50 (14.0%)	6/31 (19.4%)	6/28 (21.4%)	0.670
PSQI	7.60 ± 3.43	6.90 ± 3.51	7.36 ± 3.12	0.569
Antiviral within 72 h	28/50 (56.0%)	16/31 (51.6%)	14/28 (50.0%)	0.859
Average 24-h NRS ≥3 at follow-up	13/50 (26.0%)	3/31 (9.7%)	9/28 (32.1%)	0.096

Data are mean ± SD or n (%). Continuous variables were compared using the Kruskal–Wallis test and categorical variables using the chi-square test. Center 1, Gazi Yaşargil Training and Research Hospital, Diyarbakır (coordinating center); Center 2, Şanlıurfa Training and Research Hospital; Center 3, Şanlıurfa Mehmet Akif İnan Training and Research Hospital. NRS, numerical rating scale; DN4, Douleur Neuropathique 4; PPT, pressure-pain threshold; PSQI, Pittsburgh Sleep Quality Index; SD, standard deviation.
